# Correlation between retinal oxygen saturation and the haemodynamic parameters of the ophthalmic artery in healthy subjects

**DOI:** 10.1111/aos.15189

**Published:** 2022-05-22

**Authors:** Michal Král, Tereza Svrčinová, Pavel Hok, Tomáš Dorňák, Martina Rybáriková, Jan Mareš, Petr Kaňovský, Martin Šín

**Affiliations:** ^1^ Department of Neurology University Hospital and Faculty of Medicine and Dentistry, Palacky University Olomouc Olomouc Czech Republic; ^2^ Department of Ophthalmology University Hospital and Faculty of Medicine and Dentistry, Palacky University Olomouc Olomouc Czech Republic

**Keywords:** automatic retinal oximetry, Doppler imaging, ophthalmic artery, retinal vessel oxygen saturation

## Abstract

**Purpose:**

The aim of the study was to obtain the values of oxygen saturation in retinal vessels and ophthalmic blood flow parameters in a healthy Caucasian population and assess whether the oximetry parameters are affected by the flow rate or the vascular resistance.

**Methods:**

The spectrophotometric retinal oximetry and colour Doppler imaging (CDI) of retinal vessels were successfully performed with 52 healthy subjects (average age 29.7 ± 5.6 years). The retinal oximeter simultaneously measures the wavelength difference of haemoglobin oxygen saturation in retinal arterioles and venules. The arteriolar and venular saturation in both eyes was measured. The peak systolic (PSV) end diastolic (EDV) velocities, resistive (RI) and pulsatility (PI) indices were obtained for both eyes using CDI in the ophthalmic artery. A paired *t*‐test and two sample t‐tests were used for statistical analyses. The correlation was assessed using the Pearson coefficient correlation.

**Results:**

The mean oxygen saturation level was 96.9 ± 3.0% for the retinal arterioles and 65.0 ± 5.1% for the retinal venules. The A‐V difference was 31.8 ± 4.6%. The mean of the measured haemodynamic parameters was PSV 46.6 ± 9.4 cm/s, EDV 12.0 ± 3.5 cm/s, PI 1.68 ± 0.38 and RI 0.74 ± 0.05. No significant difference in oxygen saturation and haemodynamic parameters was found between the left and the right eyes or the dominant and non‐dominant eye. The oximetry and ultrasound values were sex independent. The Pearson correlation coefficient demonstrated a significant yet weak negative correlation between A‐V difference and RI (*r* = −0.321, p = 0.020).

**Conclusions:**

A negative correlation between A‐V difference and resistance index was observed, suggesting that reduced oxygen consumption may reflect the increased vascular tone of the ophthalmic vessels, which is likely determined by autoregulatory mechanisms.

## Introduction

The retina is a highly metabolically active tissue with a rich blood supply and the retinal vessels are relatively easily measurable from the outside (Vosborg, Malmqvist & Hamann 2020, Wei et al. 2018). Abnormal oxygen saturation in retinal vessels has been demonstrated in a number of retinal diseases and even in systemic diseases, including neurological disorders (Stefánsson et al. 2019). Retinal vessel saturation could therefore serve as a possible biomarker of many retinal and brain diseases (Stefánsson et al. 2017).

The retina can provide information about oxygen saturation in the central nervous system vessels and is a promising target for assessment of haemodynamic parameters and their correlation with the oxygen supply. The retinal vessel oximetry values have been shown to be reliable and relatively stable in healthy individuals (Geirsdottir et al. 2012, Türksever et al. 2015, Yip et al. 2014).

Despite these recent advances, further data are needed to improve our understanding of disease‐associated abnormalities and to allow for future comparisons between the healthy population and various patient cohorts. Firstly, variability among various sub‐populations should be considered when interpreting the pathological conditions (Nakano et al. 2016). Most of the normative data thus far were obtained in an Asian population (Mohan et al. 2015, Nakano et al. 2016, Yang et al. 2016), juvenile population (Liu et al. 2017, Vehmeijer et al. 2016, Waizel et al. 2018) or under specific conditions, for example in animal models while breathing 100% oxygen (Li et al. 2016, Olafsdottir et al. 2015). Only a minority of the studies focused on normal values of retinal vessel oxygen saturation in a healthy adult Caucasian population (Geirsdottir et al. 2012, Jani et al. 2014).

Secondly, there are no data up to now as to what extent the retinal oximetry measurement is influenced by the haemodynamic parameters of the ophthalmic artery circulation. Peripheral blood flow and blood oxygenation level are tightly coupled as shown in studies combining laser Doppler flowmetry with pulse oximetry during vascular occlusion challenges, both in the animal model (Hallock & Rice 2003) and in healthy individuals (Abay & Kyriacou 2016). In the ocular vessels, blood flow velocity and vascular resistance can be measured non‐invasively using the ultrasound colour Doppler imaging (CDI) of the ophthalmic artery. Despite many years of clinical use of CDI in ophthalmology, the procedure is not yet fully standardized and requires an experienced and skilled examiner (Vosborg et al. 2020). There is also a relative lack of normative retinal blood flow data that also limits the potential use of CDI in clinical practice. Most of the studies thus far have focused on ophthalmological diseases, for example diabetic retinopathy, glaucoma and age‐related macular degeneration (Dimitrova et al. 2002, Evans et al. 1997, Harris et al. 2000, MacKinnon et al. 2000), while data in the healthy population remains insufficient (Bittner et al. 2016).

Studies combining retinal oximetry and CDI are rare. In a recent study, both retinal oximetry and CDI were significantly discriminative for normal‐tension glaucoma and primary open‐angle glaucoma (Barbosa‐Breda et al. 2019). To the best of our knowledge, however, there has been no report of correlation between ophthalmic artery haemodynamic parameters (CDI) and retinal vessel oxygen saturation levels in healthy individuals. While flow parameters can be affected mainly by cardiovascular and respiratory diseases, there is a need to determine the relationship between ophthalmic circulation and retinal metabolism under physiological conditions in a healthy population.

Therefore, the first aim of the study was to determine the normal values of retinal vessel oxygen saturation together with ultrasound (CDI) parameters in ophthalmic vessels, especially the values of the blood flow velocities and vascular resistance in Caucasian population. The second aim was to assess whether the oximetry is affected by any of the ophthalmic artery CDI parameters. We hypothesized two possible scenarios: in case of no association between oximetry and CDI parameters, the finding would support the claim that retinal oximetry mainly reflects dysfunction of the retinal metabolism without interference of other vascular conditions. Mlčák et al. (2022) demonstrated in a study in patients with diabetes that oximetry values were only minimally affected by the systemic environment. Conversely, in the case of a significant relationship between oximetry and CDI values, such an association should be taken into account when interpreting the oximetry results in future studies. Additionally, oximetry could potentially be used for indirect monitoring of disorders affecting ophthalmic circulation.

## Materials and Methods

This prospective study followed the tenets of the Declaration of Helsinki. Written informed consent was obtained from all subjects before participation in this study, and the study was approved by the Ethics Committee of the University Hospital Olomouc and Palacký University Olomouc (approval number NV19‐06‐00216).

### Subjects

Fifty‐five healthy subjects (controls) were enrolled in the study at University Hospital Olomouc between January 2019 and July 2021. Three women were subsequently excluded in this group due to less than normal best‐corrected visual acuity. A total of 52 controls were enrolled, mean age 29.7 ± 5.6 years, 19 males (36.5%), 33 females (63.5%), in detail in Table 1. The exclusion criteria were a history of diabetes, arterial hypertension, severe cardiovascular and respiratory diseases, stenosis of the internal and external carotid artery, refractive errors greater or equal +/−6 dioptres, best‐corrected visual acuity less than 1.0, eye trauma, eye surgery, and eye diseases (glaucoma, cataract, keratoconus, retinal venule or arteriole occlusion). All the participants were examined according to the same study protocol: ocular examination, which included examination of visual acuity (Snellen chart), ocular dominance testing using a distance hole‐in‐the‐card test and assessment of oxygen saturation in major retinal vessels. CDI was performed within several days to evaluate ophthalmic artery haemodynamic parameters and to rule out stenosis of the main cerebral arteries.

**Table 1:** Group age distribution.

	Valid *N*	Mean	SD
Group	52	29.71	5.65
Female	33	28.09	3.87
Male	19	32.53	7.11

*N* = number, SD = standard deviation.

### Automatic retinal oximetry

Before the measurement, the pupil was dilated to a diameter of approximately 5 mm using 1% tropicamide eye drops (Mydriacyl; S.A. Alcon‐Couvreur N.V., Puurs, Belgium). Retinal oximetry was carried out using an Oxymap oximeter (Oxymap ehf., Reykjavik, Iceland) which was connected to a fundus camera (Topcon DX‐50, Topcon Inc., Tokyo, Japan) which measures haemoglobin oxygen saturation by comparing the images of the retinal vessels acquired at different wavelengths (570 nm and 600 nm). The technical details are described elsewhere (Geirsdottir et al. 2012). A standardized technique was used: participants were sitting in a dark room to avoid the influence of ambient light. Fundus photographs were taken in a 50° field centred at the temporal edge of the optic disc and the light flash was set at 50 Ws. Image analyses were performed according to a standardized protocol (Oxymap protocol for acquisition and analysis of Oxymap T1 oximetry images, version November 21, 2013; Oxymap ehf.) using the software Oxymap Analyser (version 3.1.4, Oxymap ehf., Reykjavik, Iceland). The analysis was carried out by a single blinded examiner (MR). The oxygen saturation was measured in all retinal arterioles and venules with the width greater than 8 pixels and a length greater than 100 pixels within the measurement zone. The measurement zone was defined as the space between the inner and the outer circle. The diameter of the inner circle was 1.5 times larger and the diameter of the outer circle was 3 times larger than the diameter of the optic disc. In the case of branching vessels, the parent branch was measured. If the parent branch was less than 100 pixels long, the daughter branches were measured. At vessel crossings, the distal segment was chosen unless this segment was less than 100 pixels long. In such cases, the proximal segment was measured. The retinal vessel oxygen saturation level was calculated as the sum of all retinal vessel oxygen saturation measurements multiplied by the diameter of each vessel to the fourth power, further divided by the sum of the diameter of each vessel to the fourth power.

$$=\frac{S_{1}*d_{1}^{4}+S_{2}*d_{2}^{4}+S_{3}*d_{3}^{4}+S_{4}*d_{4}^{4}+S_{5}*d_{5}^{4}+S_{6}*d_{6}^{4}+S_{7}*d_{7}^{4}+S_{8}*d_{8}^{4}}{d_{1}^{4}+d_{2}^{4}+d_{3}^{4}+d_{4}^{4}+d_{5}^{4}+d_{6}^{4}+d_{7}^{4}+d_{8}^{4}}$$

Mean oxygen saturation S = saturation of n vessel segment n d = diameter of n vessel segment.

The arterio‐venous (A‐V) difference was calculated as the difference between the retinal arteriolar oxygen saturation (AS) and the retinal venular oxygen saturation (*VS*) (Feke et al. 1989, Hardarson 2013). We used the same analysis as in our previous work where the full description can be found (Šín et al. 2016).

### Neurosonology

The CDI examination was performed using 2–8 MHz linear probe (9 L‐D, LOGIQ S8, GE Healthcare, Milwaukee, WI, USA). We followed the protocol of Modrzejewska (2019), where the participants were examined bilaterally in the supine position after a 5‐min rest. The participants were lying with their eyes closed, while both eyes were looking straight ahead or downwards. For safety reasons, the lowest possible ultrasound intensity and the shortest possible sonication times were used to avoid any damage to the eye structures. The measurement angle for the ophthalmic artery did not exceed 60° and the flow velocity parameters were taken at the intersection of the optic nerve and the artery (Modrzejewska 2019). Peek systolic velocity (PSV) and end diastolic velocity (EDV) were obtained and used to calculate the resistive index and pulsatility index in the ophthalmic artery. Resistive index (RI) indirectly determines vascular resistance and is calculated as (PSV ‐ EDV)/PSV (Planiol et al. 1973). The RI is the highest in the ophthalmic artery and may remain unchanged or increase with age in healthy individuals (Modrzejewska 2019). Pulsatility index (PI) is calculated as (PSV ‐ EDV)/V_mean_, where V_mean_ = 1/3 (PSV/EDV) + EDV (Gosling & King 1974). Similar to RI, PI is the highest in the ophthalmic artery and increases with age. Additionally, the internal and external carotid arteries on both sides were examined in all subjects to detect possible stenosis that may influence the results. Extracranial haemodynamic parameters were neither collected nor further analysed.

### Statistical analysis

The statistical analysis was performed using the software STATISTICA 12 (https://www.statistica.pro/). All quantitative data were expressed as mean, standard deviation (SD), minimum and maximum. Boxplots were used for visual comparison of the samples. A paired *t*‐test was used to distinguish between two paired sets of data (right *vs* left eye and dominant *vs* non‐dominant eye). The values from both eyes were averaged within subject for the remaining analyses. Two sample *t*‐tests were used to distinguish between groups by gender. Relationships between CDI and oximetry parameters were measured by the Pearson coefficient of correlation. Scatterplots were used for a visual comparison of the relationships between the parameters. All the tests were performed at the 5% level of significance.

## Results

### Overall distribution

A total of 104 eyes were examined. The mean oxygen saturation level was 96.9 ± 3.0% for the retinal arterioles, 65.0 ± 5.1% for the retinal venules. The A‐V difference was 31.8 ± 4.6%. The mean of the measured haemodynamic parameters was PSV 46.6 ± 9.4 cm/s, EDV 12.0 ± 3.5 cm/s, PI 1.68 ± 0.38 and RI 0.74 ± 0.05. This can be seen in detail in Table 2.

**Table 2:** Descriptive statistical data for overall distribution in both eyes.

Parameter	Number	Mean	SD
PSV [cm/s]	104	46.65	9.43
EDV [cm/s]	104	12.00	3.59
PI	104	1.69	0.38
RI	104	0.74	0.06
AS [%]	104	96.91	3.08
*VS* [%]	104	65.05	5.14
A‐V difference [%]	104	31.87	4.62

AS = arteriolar saturation, A‐V = arterio‐venous, EDV = end diastolic velocity, PI = pulsatility index, PSV = peak systolic velocity, RI = resistance index, SD = standard deviation, *VS* = venular saturation.

### Eye laterality

The paired *t*‐test was used to assess the statistical significance of the difference between the right and left eyes. There were no statistically significant results for either retinal vessel oxygen saturation or ophthalmic artery haemodynamic parameters, see Table 3.

**Table 3:** Comparison of ultrasound and oximetry parameters by eye laterality.

Parameter	Right eye (*n* = 52)		Left eye (*n* = 52)		p (paired *t*‐test)
	Mean	SD	Mean	SD	
PSV [cm/s]	46.65	9.70	46.65	9.26	0.998
EDV [cm/s]	12.24	3.86	11.77	3.31	0.158
PI	1.66	0.34	1.72	0.42	0.221
RI	0.74	0.06	0.75	0.06	0.335
AS [%]	96.96	3.11	96.87	3.07	0.855
*VS* [%]	64.92	5.82	65.17	4.41	0.746
A‐V difference [%]	32.04	5.16	31.69	4.06	0.611

A‐V = arterio‐venous, EDV = end diastolic velocity, PI = pulsatility index, PSV = peak systolic velocity, RI = resistance index, SD = standard deviation, *VS* = venular saturation.

### Eye dominance

The paired *t*‐test was used to assess the statistical significance of the difference between the dominant and non‐dominant eye. It is apparent that no parameter was statistically significant, see Table 4. The tree subjects for whom eye dominance was not determined were excluded from the analyses.

**Table 4:** Comparison of ultrasound and oximetry parameters by eye dominance.

Parameter	Dominant eye (*n* = 49)		Non‐dominant eye (*n* = 49)		p (paired *t*‐test)
	Mean	SD	Mean	SD	
PSV [cm/s]	46.60	10.32	47.16	8.87	0.576
EDV [cm/s]	12.31	4.01	11.76	3.19	0.114
PI	1.65	0.36	1.72	0.42	0.223
RI	0.74	0.06	0.75	0.06	0.097
AS [%]	97.04	2.63	96.94	3.37	0.849
*VS* [%]	65.02	5.99	65.35	4.39	0.682
A‐V difference [%]	32.02	5.43	31.59	3.85	0.550

AS = arteriolar saturation, A‐V = arterio‐venous, EDV = end diastolic velocity, PI = pulsatility index, PSV = peak systolic velocity, RI = resistance index, SD = standard deviation, *VS* = venular saturation.

### Gender

The resulting values of the two sample t‐tests indicate that the groups do not significantly differ statistically in any of the monitored parameters, see Table 5.

**Table 5:** Comparison of ultrasound and oximetry parameters by gender.

Parameter	Males (*n* = 19)		Females (*n* = 33)		p (two sample *t*‐test)
	Mean	SD	Mean	SD	
PSV [cm/s]	48.10	11.43	45.82	6.85	0.372
EDV [cm/s]	13.00	4.06	11.43	2.86	0.109
PI	1.69	0.36	1.69	0.34	0.996
RI	0.73	0.05	0.75	0.05	0.246
AS [%]	96.45	2.39	97.18	2.47	0.301
*VS* [%]	63.68	4.53	65.83	4.12	0.087
A‐V difference [%]	32.76	3.99	31.35	3.89	0.217

AS = arteriolar saturation, A‐V = arterio‐venous, EDV = end diastolic velocity, PI = pulsatility index, PSV = peak systolic velocity, RI = resistance index, SD = standard deviation, *VS* = venular saturation.

### Correlation of oximetry and ophthalmic artery haemodynamic parameters

The dependences between the observed ultrasound and oximetry parameters were assessed using Pearson correlation coefficients arranged in a correlation matrix as the data met the assumption of normality and did not contain extreme outliers.

**Fig. 1:**
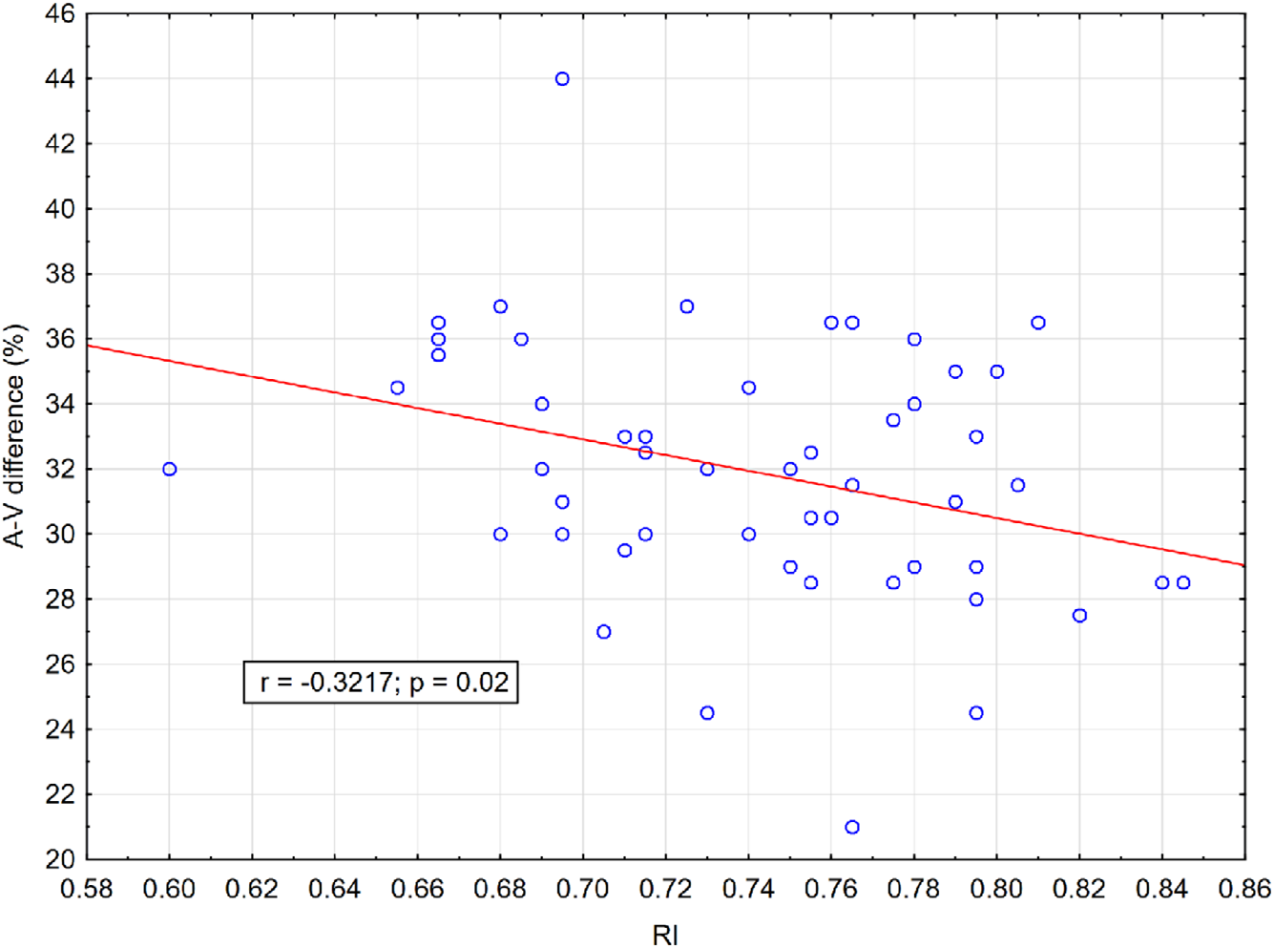
Correlation of average values. The figure shows a scatter plot illustrating the relationship between arterio‐venous (A‐V) difference (ordinate) and resistance index (RI, abscissa) for the within‐subject averaged data (*N* = 52). The figure includes Pearson's correlation coefficient (*r*) and p value as well as the least squares regression line. [Colour figure can be viewed at wileyonlinelibrary.com]

The analysis yielded a statistically significant yet weak negative correlation between A‐V difference and RI (*r* = −0.321, p = 0.020), and a similar trend was observed between A‐V difference and PI (*r* = −0.257, p = 0.065), see Fig. 1.

## Discussion

To date, there is no gold standard for the assessment of the blood flow and oxygen saturation in the retinal vessels. The development of new diagnostic methods or the use of established methods in a new field or combination of both can uncover new biomarkers and bring new perspectives into clinical practice (Vosborg et al. 2020, Wei et al. 2018).

The first aim of this study was to evaluate oxygen saturation in retinal vessels and the ophthalmic artery haemodynamic parameters in healthy subjects of Caucasian descent. The second aim was to determine whether the oximetry is affected by the results of ultrasound measurement.

### Retinal oximetry

Retinal oximetry is an innovative method that allows for the assessment of retinal vessel oxygen saturation, provides information about oxygen consumption in tissues and introduces new potential biomarkers of retinal diseases. It also seems to be a promising method to determine new biomarkers in systemic and brain diseases (Olafsdottir et al. 2018, Stefánsson et al. 2019, Svrčinová et al. 2020). The reproducibility of oximetry is excellent; however, in some cases (*e.g*. in glaucoma and inherited retinal disease) it seems more reliable in arterioles than venules (Türksever et al. 2015).

Previous studies have suggested that retinal oximetry is superior in determining oxygen transport to the brain than peripheral pulse oximetry (Eliasdottir 2018). The disadvantage of current retinal oximetry applications (*e.g*. Oxymap) is the lack of a built‐in normative database; researchers have to create their own standard normative database. The oximetry data are very stable; however, the average standard deviation during retesting of the same participant is only 1% (Stefánsson et al. 2017).

Since 2012, several studies examining oxygen saturation in healthy individuals have been published. According to the results of previous studies, retinal vessel oxygen saturation is affected by age, gender and ethnicity (Geirsdottir et al. 2012, Jani et al. 2014, Mohan et al. 2015, Nakano et al. 2016, Palsson et al. 2012, Waizel, Türksever & Todorova 2018, Yip et al. 2014).

In the current study, 104 healthy eyes were examined. No significant changes between the left and right eyes were observed. The first work about retinal vessel oxygen saturation in a healthy adult population was published by Geirsdottir et al. (2012). The authors investigated 120 controls, all Caucasians, aged 18–80 years (median 47 years). In this large cohort, the mean oxygen saturation was 92.2 ± 3.7% in arterioles and 55.6 ± 6.3% in venules (Geirsdottir et al. 2012).

Jani et al. (2014) reported normative values and predictors in 61 controls of mixed ethnicity, aged 19–74 years (mean age 44.1 ± 14.7 years). The mean oxygen saturation was 90.4 ± 4.73% in arterioles and 55.3 ± 7.1% in venules. Reliability of retinal vessel oximetry in an Asian healthy population was evaluated by Yip et al. (2014). In that study, 118 participants were investigated, all being older than 40 years, the mean AS 93.64 ± 6.9% and the mean *VS* 54.22 ± 6.9%. Another study in healthy adults was reported by Mohan et al. (2015). In 30 Indian subjects aged 18–63 years (mean 33), the mean AS was 90.3 ± 6.6% and the mean *VS* was 56.9 ± 6.3%. In the Japanese population, Nakano et al. (2016) reported the mean AS 97.0 ± 6.9% and the mean *VS* 52.8 ± 8.3% in 252 healthy subjects aged 20–93 years (mean 61).

In the current study, relatively higher values of saturation in retinal venules were observed compared with the previous studies. This can be due to several reasons. Firstly, it might be the result of our focus on younger healthy adult individuals (mean 29.7 years). Similar results were reported in the study of Palsson et al. (2012). In their study group, 26 healthy individuals aged 18 to 30 years were included, yielding a mean AS 93.1 ± 2.3% and a mean *VS* 64.9 ± 3.3%. One possible explanation for the increased retinal vessel oxygen saturation in the young population may be increased opacity of the eye medium in the elderly (lens, cornea, vitreous and aqueous humour). The opacities in the ocular medium can cause wavelength changes and alter the accuracy and reproducibility of the oximetry (Chen et al. 2017, Della Volpe Waizel et al. 2020, Šín et al. 2016). A similar explanation was suggested by Geirsdottir et al. (2012) who also proposed the possible role of impaired pupil dilatation in older individuals. These factors should have a minimal influence in our young population. In a study in a paediatric population, Waizel et al. (2018) found that children between the ages of 10 and 20 had significantly higher VS than the adult population. In general, the authors concluded that children had mainly lower AS and A‐V difference, which has been linked to greater overall metabolic and growth activity and lower oxygen utilization in the neuronal tissue in childhood.

We also considered the possibility that we tested a single subgroup of the Caucasian race, namely a Slavic population. Retinal oximetry is based on measurement of haemoglobin wavelength difference in retinal vessels, while it measures changes in wavelength against a relatively constant ocular background. Hence, changes in cross‐race measurements may be related to pigment changes in the ocular background (Bourne 2011, Rochtchina et al. 2008).

### Ophthalmic artery haemodynamic parameters

The haemodynamic parameters were investigated in 104 ophthalmic arteries. According to a review of the use of CDI in assessing ocular blood flow (Stalmans et al. 2011), our PSV, EDV values are slightly higher than in several previous studies. This may be due to the different design (sex, age and comorbidities) of the assessed studies in Stalmans' review and differences in the population and ethnic composition. Other studies, however, reported similar results to ours (Querfurth et al. 2002, Repo et al. 1995, Rojanapongpun et al. 1993, Stalmans et al. 2009, Tranquart et al. 2003). In the Leuven study, early systolic acceleration (ESA, that is the slope of the fastest‐moving portion of the systolic wave) and EDV in the ophthalmic artery were evaluated in patients either with open‐angled glaucoma or normal‐tension glaucoma. ESA significantly differed between the groups, whereas EDV was not discriminative (Barbosa‐Breda et al. 2019). Although we did not evaluate ESA in our work, as it is not a commonly used CDI parameter, it would be appropriate to take it into account in future studies.

### The relationship between oximetry and CDI

To the best of our knowledge, this is the first study to investigate the relationship between the ophthalmic haemodynamic parameters and the saturation parameters in the retinal vessels in a healthy Caucasian population. In the Leuven study, an advanced vascular examination (CDI, retinal oximetry, ocular pulse amplitude and choroidal thickness) was assessed in patients with glaucoma, but correlations between the retinal vessel oxygen saturation and ultrasound characteristics were not assessed (Barbosa‐Breda et al. 2019).

The retina is highly metabolically active; it requires the highest supply of oxygen per gram of tissue in the human body. The circulation in the retina has, compared with other tissues, low blood flow and a high extraction ratio of oxygen (Rassam et al. 1996). The retina has several mechanisms of autoregulation of blood flow parameters and oxygen delivery. The first mechanism is the mechanical regulation of the blood flow (*via* vasodilation and vasoconstriction), which is controlled biochemically (Pournaras et al. 2008). The RI seems to be the most reliable parameter of normal circulation in ophthalmic arteries reflecting the blood flow (Kouvidis et al. 2000). Although we did not detect any relationship between the ophthalmic blood flow velocity parameters and oxygen saturation either in retinal arterioles or venules, we observed a negative correlation between RI and A‐V difference, which is an indirect marker of oxygen consumption. A similar trend was observed when examining the relationship between PI and A‐V difference, but it did not reach a statistical significance. Reduced oxygen consumption (A‐V difference) may therefore reflect increased vascular tone (resistance) of the ophthalmic vessels. We assume that vascular resistance in our cohort was mainly determined by autoregulatory mechanisms as no participant was treated for any systemic vascular disease and sub‐clinical disease is unlikely in young adults. This supports the theory that autoregulation is achieved by adaptation of the vascular tone of the resistance vessels. Retinal vascular endothelial cells are able to release factors, which actively change the tone of the blood vessel walls and subsequently the release of oxygen (Pournaras et al. 2008).

Our finding of a negative correlation between RI and A‐V difference may seem counter‐intuitive in light of the claim that RI gradually increases with age (Modrzejewska 2019) while A‐V difference also increases up to age of 30–40 (Waizel et al. 2018). However, A‐V difference is showing non‐linear age dependence as it is decreasing after the age of 40 (Waizel et al. 2018). According to Waizel et al. (2018), gradually increasing oxygen utilization in the neuronal tissue and decreasing metabolic demands of other tissues in children and young adults underlie the increasing A‐V difference in retinal vessels. Hence, the relatively weak negative correlation between the RI and A‐V difference observed in our cohort might simply be overshadowed by these age‐related mechanisms. With ageing, different processes may dominate the changes in A‐V difference. Jeppesen et al. (2004) observed a decrease in retinal vessel autoregulation in persons over 40 years of age. This may be associated with gradually decreasing A‐V difference at this age, although causal relationship is yet to be proved. Our findings in a relatively homogeneous cohort of young adults cannot be easily extrapolated to different age categories, but a stronger relationship between the RI and A‐V difference in different age categories cannot be ruled out and should be considered in future studies.

There are certain limitations of CDI that should be acknowledged. The interpretation of RI and its relationship to vascular resistance in the ophthalmic circulation is still controversial (Modrzejewska 2019, Stalmans et al. 2011). The RI values in the ophthalmic artery and in the central retinal artery have shown inconsistent age dependence in different cohorts (Harris et al. 2000, Modrzejewska et al. 2015). Studies that would include CDI in both the ophthalmic artery and the central retinal artery are thus warranted.

Based on our results, CDI of the ophthalmic artery was able to explain some yet relatively small proportion of the variability in the A‐V difference in our cohort of relatively young healthy adults. This relationship should be further explored, however, in both younger and older populations, as well as in patient cohorts, in which vascular or haemodynamic abnormalities can be expected.

## Conclusions

In conclusion, retinal vessel oxygen saturation levels and ophthalmic artery haemodynamic parameters in a young Caucasian population are in general agreement with values in other populations. While there was no correlation between oximetry parameters and velocity blood flow parameters, a negative correlation between A‐V difference and resistance index was observed, suggesting that reduced oxygen consumption may reflect the increased vascular tone of the ophthalmic vessels, which is likely determined by autoregulatory mechanisms.
